# A Method Considering Multi-Dimensional Feature Differences for Extracting Rural Buildings Based on Airborne LiDAR

**DOI:** 10.3390/s26020652

**Published:** 2026-01-18

**Authors:** Siyuan Xi, Jianghong Zhao

**Affiliations:** 1 School of Geomatics and Urban Spatial Information, Beijing University of Civil Engineering and Architecture, Beijing 100044, China; 1108160025007@stu.bucea.edu.cn; 2 Engineering Research Center for Representative and Ancient Building Database of the Ministry of Education, Beijing 100044, China

**Keywords:** building classification, feature difference, rural area, airborne lidar

## Abstract

**Highlights:**

**What are the main findings?**

- A framework comprising ground point classification, building Region of Interest filtering, and refined extraction is proposed based on airborne LiDAR data for building classification purposes in complex rural scenes.
- Ground points form the foundation for building classifications. Region of Interest filtering based on geometric features further confirms building scopes, then a set of morphological features primarily based on local dimensionality models enables precise building classification.

**What are the implications of the main findings?**

- The proposed framework employs a spatial hierarchical strategy to extract building data, thereby avoiding the substantial redundant computations caused by traversing the entire point cloud. By precisely capturing local features of point clouds within an optimal neighborhood range, it achieves high-precision building classification.
- This work provides a comprehensive approach for point cloud recognition of low-rise structures in rural areas, particularly suited for regions where vegetation and buildings are of similar height and exhibit an interlocking pattern. It demonstrates significant potential for use with other intelligent building classification methods.

**Abstract:**

Research on extracting building from airborne point clouds is abundant, yet discussions regarding scenarios where vegetation and building structures are closely intertwined with similar height in rural areas remain relatively scarce. This thesis adopts a region representative of typical rural building features in China as an experimental site to conduct research on building classification procedures from airborne point clouds. Firstly, the multi-level grid size is dynamically determined through slope analysis to creatively segment and recognize terrain type, then differentiated filtering parameters are applied to various terrains to fully extract ground points, providing a ground reference for building classification. Secondly, the selection of building Region of Interest is conducted by multiple geometric feature differences between building and other objects based on watershed segmentation results, which eliminates interference from non-building points, significantly reducing redundant and unnecessary mathematical computation. Finally, refined building classification is achieved based on multiple morphological differences between buildings and other objects. The experimental results show that the precision, recall, and F1 of both datasets exceeded 93.37%, 97.05%, and 95.17%, respectively. The average precision, recall, and F1 reached 94.02%, 97.20%, and 95.58%, respectively. This method demonstrates successful building classification in rural areas, showing strong adaptability and practicality for the extraction of various building data.

## 1. Introduction

Light Detection and Ranging (LiDAR) is a measurement system integrating global positioning technology, inertial navigation technology, and laser ranging technology, enabling rapid, high-precision, real-time acquisition of three-dimensional surface information [1]. Building classification procedures based on LiDAR point clouds enables rapid acquisition of spatial distribution, area, height, volume, and other attribute information for large-scale groups of buildings, and holds significant importance across diverse fields including urban planning, disaster damage assessment, and socioeconomic surveys [2]. In practical applications, building point clouds provide foundational data for urban planning, management, and city map updates. This enables city administrators to gain a comprehensive understanding of the city’s operations, thereby facilitating more effective decision-making to enhance urban operational efficiency. Additionally, building classification based on airborne LiDAR can determine the location, extent, and scale of disaster-affected structures, monitor the dynamic changes, assess disaster progression trends, and provide a scientific basis for emergency rescue operations, such as determining rescue routes and evaluating disaster losses. It also supplies foundational data for post-disaster reconstruction, including planning reconstruction and assessing reconstruction feasibility.

The building classification process based on airborne LiDAR point clouds primarily consists of two steps: ground point filtering and building classification. The purpose of point cloud filtering is to accurately separate ground points from disordered point clouds, restoring topographical relief, and subsequently extracting building points from non-ground points. Common methods for point cloud filtering include mathematical morphology filtering [3], progressive triangulated irregular network densification filtering [4] (PTD), and cloth simulation filtering [5] (CSF). Most filtering methods perform well in areas with continuous terrain, good penetration, gentle slopes, and low vegetation coverage. Generally speaking, it is hard for regions with discontinued terrain, steep slopes, and poor penetration to achieve great results. Zheng et al. [6] adopted an outlier removal method to establish an adaptive two-level grid, then used an adaptive selection method for angle and distance thresholds in the iterative densification processing. Nie et al. [7] revised the classic PTD method by building an improved triangulated irregular network (TIN) and changing the original iterative judgment criteria for better filtering of airborne LiDAR point clouds. Cai et al. [8] used CSF and PTD together, obtaining a high-quality initial provisional digital terrain model (DTM) via CSF, and the parameter thresholds of PTD were estimated from the initial provisional DTM based on statistical analysis theory. Finally, PTD with adaptive parameter thresholds is used to refine the initial provisional DTM. However, applying the same single filtering parameter for all the above methods makes it difficult to reach an optimal result, especially in mountainous areas with greater vegetation coverage. Therefore, exploring ground point filtering methods that adapt to complex terrain is a current hot topic.

Regarding building classification methods, early researchers adopted the segmentation approach to extract building information based on raster images derived from point cloud sources. Li et al. [9] generated a Normal Digital Surface Model (NDSM) from point clouds, and a transformation of the sign watershed was conducted while controlling for both height and gradient to obtain ground objects. Finally, using built feature indicators, building objects were identified based on a maximum likelihood classification. Wu et al. [10] used morphological methods to perform dilation and erosion on the Digital Surface Model (DSM) to classify buildings. These methods for converting 3D point clouds into 2D images establish topological relationships between grids but cannot directly express the topological relationships between isolated points, sacrificing and overlooking the accuracy and detail of the original point cloud. Subsequently, with the advent of semantic segmentation, this method has also been applied to building classification. Li [11] estimated normal vector differences between each point and the cluster ground, taking projection density characteristics of the point cloud and region growth algorithm to separate building points from the non-ground points. Su et al. [12] used CSF to identify non-ground points, followed by regional growth for coarse building classification. Finally, they integrated the Alpha Shape algorithm with neighborhood expansion methods to refine the coarse-extracted building point cloud. Studies on the above two types of traditional approaches have made significant progress, but there remains room for improvement in handling complex scenarios.

With the widespread adoption of machine learning (ML), these approaches have also been applied to building classification methods. ML algorithms rely on a pre-trained model on given data consisting of inputs and their corresponding outputs with the same characteristics. A small group of point cloud representatives is selected for training, in order to obtain a classifier, which is designed to classify buildings accurately. Common classifiers contain support vector machines [13], random forests [14], naive bayes [15], etc. Morsy et al. [16] assessed the relevance of sixteen LiDAR-derived geometric features, and applied the most contributing features to the classification process. Then, a pointwise classification method based on random forests is applied to extract building data. Chen et al. [17] used random forest to extract building data at the pixel level, then implemented image segmentation through a super-pixel segmentation algorithm. Yang et al. [18] introduced a supervised sparse coding-enhanced bag of visual word (SC-BOVW) model based on a learned discriminative dictionary to encode local geometric and spectral information within each super-voxel into high-level semantic representation, which was then fed into a support vector machine (SVM) classifier to distinguish buildings from each other. Kaplan et al. [19] designed a comprehensive method to combine random forest, random tree, and optimized forest classifiers to extract building data, and achieved a high accuracy result. Machine learning heavily relies on the selection of features and classifiers, and requires manual feature design for algorithms, showing poor generalization capabilities, which limits its flexibility.

In recent years, some researchers integrated deep learning (DL) with point cloud classification. Based on model learning, they enable autonomous learning of high-level abstract features to form a complete deep learning model, thereby implementing target recognition and segmentation. Common neural networks include convolutional neural networks [20], PointNet++ [21], and transformer [22], etc. Zhu et al. [23] designed novel convolutional operators based on the disordered and unstructured nature of point clouds. By constructing convolutional neural networks, they directly learned about multi-level features from unstructured point clouds and obtained category labels for each point in an end-to-end manner, thereby achieving point cloud classification. Shin et al. [24] proposed a method for semantic segmentation and building classification using PointNet++ to process multi-echo data from airborne LiDAR. This approach fully considers the characteristic of laser pulses making two returns at building boundaries, further enhancing building classification accuracy. Wang et al. [25] proposed a semi-supervised building classification framework. First, they generated weak image samples of a building based on unsupervised classification results of LiDAR point cloud data. Then, an iterative optimization strategy for the weak samples is proposed to compare and analyze the weak samples with the real samples and separate the accurate samples from the weak samples. Finally, the real samples, the weak samples, and the optimized weak samples were input into the semantic segmentation model applied to the building, for accuracy evaluation and analysis. Applying deep learning to point cloud classification leads to three main challenges. First, the irregular and disordered nature of point cloud data causes existing neural networks to be incompatible with convolutional operations. Second, the extraction of both local and global features from point clouds remains insufficiently comprehensive. Finally, DL requires a large number of training samples to achieve better classification performance, and the models are more sensitive to error patches in the training samples. These three factors prevent deep learning-based point cloud classification from achieving high accuracy and efficiency simultaneously. In the future, deep learning needs to evolve toward lightweight approaches while also incorporating the strengths of other traditional methods. As computing ability continues to advance, deep learning will become the mainstream approach.

The essence of the building classification process is to group point clouds according to the greatest difference in target object and find out which classification features are suitable for the target object. Despite the fact that the recently proposed methods mentioned above have significantly improved the performance of building classification, the main challenges still need to be addressed. These include the following:

(1) Ground points form the foundation for building classification. When facing the terrain in a complex scenario, single filtering parameters struggle to extract ground points fully and accurately.
(2) Raster image segmentation and classification methods primarily rely on geometric information from fixed neighborhoods within point clouds, which features a relatively single structure and only considers certain types of features. Consequently, it is susceptible to interference from surrounding objects and is unsuitable for the precise extraction of building information.
(3) For both machine learning and deep learning processes, classification accuracy heavily relies on the variety and quantity of high advanced features, causing both to be unsuitable for building classification in large-scale complex scenes.
(4) Most current methods primarily focus on classifying buildings in urban areas, with limited research conducted in rural regions. Existing building classification methods in rural areas often require traversing the entire point cloud to extract high-level abstract features, neglecting the spatial distribution and features of the buildings themselves. Additionally, the heights of buildings and vegetation canopies are very similar, making building classification somewhat challenging. These lead to redundant computations and unsatisfactory building classification results.

To overcome the above problems, this paper proposed a building classification method that makes full use of multi-dimensional feature differences. The main contributions of the proposed method are the following four aspects:

(1) The ground is the foundation for building classification. A new method entitled progressive triangulated irregular network TIN densification filter [26] based on terrain awareness (PTDTA) was proposed to extract ground points, and identifies various terrain types, adopts different filter parameters in different terrain types, and reduces errors caused by single filtering parameters.
(2) Based on watershed segmentation results after Gaussian smoothing [27], we utilized a set of geometric feature differences to pick up Region of Interest (ROI) data, excluding non-building points and reducing unnecessary computation.
(3) The local dimensional feature probability model under the restriction of minimum entropy is proposed to obtain the probability of a single point within ROI falling into different dimensions, thus optimizing the building classification procedure.
(4) Comprehensively considering the obvious planar feature of buildings, planar-related morphological feature differences are used to achieve precise building classification from the ROI.

The method proposed pays attention to the spatial distribution characteristics of buildings and focuses on the fundamental feature differences between buildings and vegetation, and effectively distinguishes buildings from vegetation.

## 2. Methods

In this section, the logic of the proposed approach is described in detail. Firstly, the overall architecture is outlined. Then, the proposed methods are separately elaborated upon.

### 2.1. Overall Architecture

**Figure 1:**
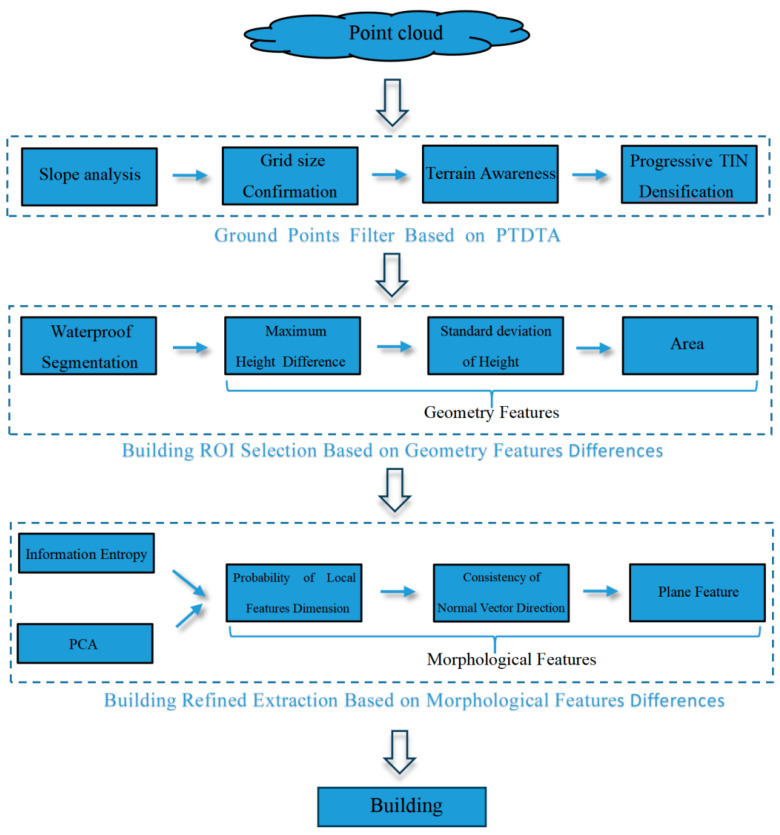
Overview of the method.

As shown in Figure 1, we divided the proposed building classification method into 3 components:

(1) Ground point filters are based on PTDTA, which implements terrain recognition according to the multi-level dynamic grid and slope, then adopts different filtering parameters to extract ground points for various terrains, thereby laying the foundation for building classification.
(2) Building ROI selection employs geometric feature difference indicators including maximum height difference and standard deviation of height and the projected area to identify buildings’ ROI, achieving a coarse pick-up of the building.
(3) Building refined extraction: This section integrates a local dimensional feature probability model, normal vector consistency, and Random Sample Consensus plan fitting [28] to complete the building classification process.

### 2.2. Ground Point Filters Based on PTDTA

To overcome the shortcomings of classic PTD, we fully take advantage of PTD strengths in reserving terrain details and adapting diverse terrain. Table 1 categorizes the terrain into three types: flat, hilly, and mountains. The extensive slope range is customized based on Reference [29] and data processing experience, which is exclusive to parameter sensitivity experiments. Finally, PTD adopts different filtering parameters for various terrains to extract ground points.

Generally speaking, the smaller the grid size, the more accurately seed points are selected, and the terrain slope better reflects terrain type. Therefore, dynamically determining grid size based on slope ensures representativeness of seed point and avoids redundant slope computations caused by overly dense grids. Terrain awareness and ground point filtering data based on multi-level grid are as follows:

(1) The point cloud is uniformly subdivided based on a primary grid. The lowest point within each grid serves as the ground seed point, and the average slope of each seed point is calculated according to Equation (1).

(1) $$Slope=\frac{\sum_{i=1}^{n}\frac{z_{i}-z_{0}}{\sqrt{{\left(x_{i}-x_{0}\right)}^{2}+{\left(y_{i}-y_{0}\right)}^{2}}}}{n}$$

where *Slope* means average slope of each seed point, (*x*_0_, *y*_0_, *z*_0_) represents the coordinates of the seed point within the target grid, (*x_i_*, *y_i_*, *z_i_*) denotes the coordinates of the seed point in the adjacent grid, and *n* indicates the quantity of adjacent grids, with a maximum value of 8.

**Figure 2:**
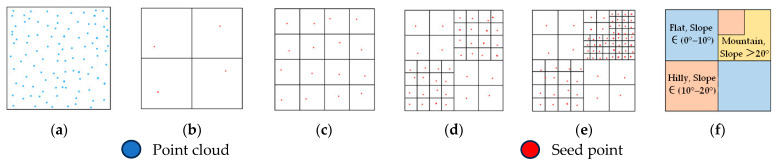
Procedure of terrain awareness: (**a**) Original point cloud; (**b**) primary grid; (**c**) secondary grid; (**d**) grid level 3; (**e**) grid level 4; (**f**) terrain awareness.

(2) The primary grid is subdivided into a secondary grid. If the average slope of seed points within both the primary and secondary grids falls within the same slope range and terrain type, it indicates that the current grid resolution is sufficiently detailed, and thus the subdivision of the secondary grid ceases. Otherwise, it indicates significant terrain fluctuation within that grid, so the secondary grid is further subdivided, generating grid level 3.
(3) Following the above procedure, we sequentially compare the average slopes of seed points within the secondary grid and grid level 3. If they fall within the same slope range, grid level 3 ceases subdivision; otherwise, subdivision continues, generating grid level 4.
(4) Through the slope adjudgment of seed points across multi-level grids, the area was divided into distinct terrain types by grids in diverse sizes, realizing terrain awareness. Based on the current grid size, the PTD was applied with different filtering parameters for each terrain type to achieve precise extraction of ground points. Figure 2 shows the complete procedure of terrain awareness.

To ensure that the secondary grid size is sufficient to encompass the minimum side length of the building and prevent the building from being incorrectly classified as ground due to an overly small grid size, the initial grid side length should be no less than twice that of the secondary grid.

### 2.3. Coarse Extraction of Building Data Based on Geometric Feature Differences

The core philosophy of coarse extraction of building data is to consider obvious geometric differences between a building and a tree, picking up the candidate area of the building from the watershed segmentation results. In this paper, geometric features including maximum elevation difference, elevation standard deviation, and area are used as a filter threshold for determining building ROI, thereby achieving coarse extraction of building data.

#### 2.3.1. Watershed Segmentation Based on Normal Digital Surface Model

The watershed segmentation treats each local minimum and its surrounding region as a catchment basin. It connects spatially adjacent pixels with similar grayscale values to form a closed basin boundary, which constitutes the watershed boundary. To avoid the impact of terrain undulations on segmentation results, this paper performs elevation normalization on the DSM, obtaining NDSM. Point clouds in rural areas feature abundant vegetation and significant elevation variations, exhibiting characteristics consistent with a Gaussian distribution. To avoid over-segmentation or under-segmentation, we use Equation (2) to smooth the NDSM prior to segmentation.

(2) $$G\left(x,y\right)=\frac{1}{2\pi \sigma^{2}}e^{-\frac{{\left(x-\mu \right)}^{2}+{\left(y-\mu \right)}^{2}}{2\sigma^{2}}}$$

where G (*x*,*y*) represents the weight of pixel (*x*,*y*), σ represents the smoothing factor, which determines the smoothing degree of image, with σ values being typically greater than 1. μ denotes the mean, typically set to 0.

#### 2.3.2. Building Interest Region Filtering

Near-ground point clouds primarily consist of buildings and vegetation, which exhibit significant differences in spatial distribution and geometric features, as detailed below:

(1) Maximum height difference is defined as the vertical distance between the highest and lowest points within a given boundary. Rural buildings are typically low, generally not exceeding three stories, resulting in a small maximum height difference. In contrast, some mature vegetation exhibit a larger maximum height difference. The method for calculating the maximum height difference is shown in Equation (3).
  (3) $$△H_{\max}=H_{\max}-H_{\min}$$
  where Δ*H*_max_ is the maximum height difference, *H*_max_ is the maximum height within the segmented area, and *H*_min_ is the minimum height within the segmented area.
(2) Standard deviation of height represents the standard deviation of all pixel elevations within a segmentation area. Building roofs are flat generally, exhibiting minimal elevation variation; in contrast, vegetation features prominent elevation differences. The method of standard deviation of height is shown in Equation (4).
  (4) $$H_{\mathrm{SD}}=\sqrt{\frac{\sum_{i=1}^{m}{\left(z_{i}-\bar{z}\right)}^{2}}{m}}$$
  where *H*_SD_ represents the standard deviation of height, *z_i_* is the elevation of *i*th pixel, $\bar{z}$ is the average elevation of pixels within the segmentation range, and *m* is the number of pixels.
(3) The projected area of the segmentation zone: Typically, building areas generally exceed vegetation canopy areas.

**Figure 3:**
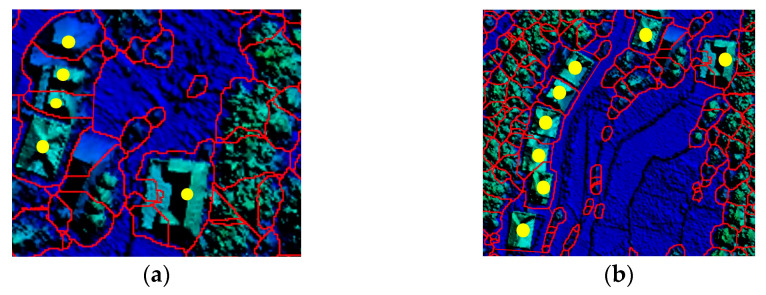
Effect of building ROI filtering: (**a**) ROI selection of sparse buildings; (**b**) ROI selection of dense buildings.

To ensure the integrity of the building, the selection should be expanded outward in a scattered pattern centered on the ROI. As shown in Figure 3, the partial area marked with yellow points represents the building ROI.

### 2.4. Refined Extraction of Building Data Based on Morphological Feature Differences

Building roofs are typically flat or sloped, exhibiting distinct two-dimensional planar Features. Vegetation canopies grow freely in all directions, exhibiting irregular three-dimensional spherical features. Consequently, the issue of precise building classification transforms into analyzing local dimensional features of point clouds, which means classifying points with obvious two-dimensional planes into building groups. At the same time, the planar features of buildings extend to the consistent direction of point cloud normal vectors. Therefore, we comprehensively consider the morphological feature differences above to reach a refined method of building classification. To enhance classification efficiency, point clouds require height filtering prior to morphological feature analysis to eliminate low vegetation near the ground, thereby further reducing computational load.

#### 2.4.1. Local Dimensional Features Probability Model

**Figure 4:**
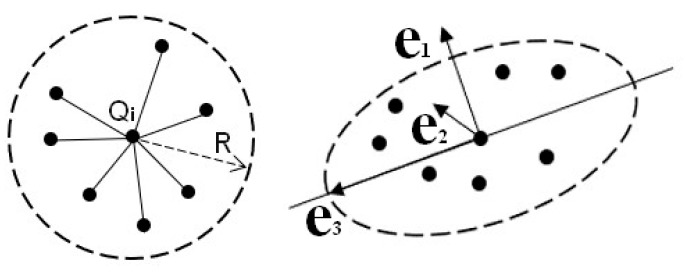
Schematic diagram of PCA: where *Q_i_* is the target point, *R* represents radius, and *e*_1_, *e*_2_, *e*_3_ represents three estimated normal vectors of the target point.

Although there is no explicit geometric–topological relationship between points, the local dimensionality features of point clouds can be described by the eigenvalues and eigenvectors of local neighboring points. Common point cloud neighborhood partitioning methods primarily include K-nearest neighbors [30] and spherical neighborhoods [30]. In comparison, spherical neighborhoods provide more accurate dimensionality features of point cloud [31]. The eigenvalues (λ_1_, λ_2_, λ_3_) and eigenvectors (e_1_, e_2_, e_3_) can be obtained by constructing the neighborhood covariance matrix using principal component analysis (PCA) [32], as shown in Figure 4.

**Figure 5:**
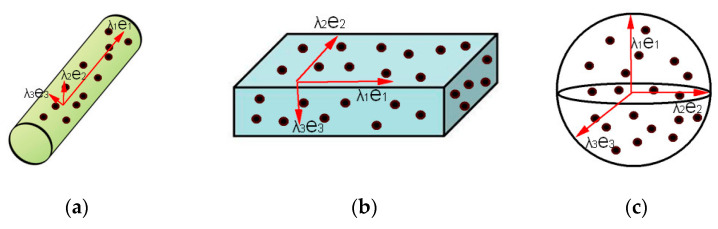
The relationship between eigenvectors and local dimensional features of points: (**a**) one-dimensional linearity; (**b**) two-dimensional plane; and (**c**) three-dimensional sphere.

At this point, the neighborhood points can be represented in a spatial cartesian coordinate system composed of the eigenvectors (*e*_1_, *e*_2_, *e*_3_) and eigenvalue (*λ*_1_, *λ*_2_, *λ*_3_). Within this new coordinate system, the value of the eigenvalues reflects the dispersion of neighborhood points across different directions. Larger eigenvalues indicate greater concentration of neighborhood points along the corresponding eigenvector direction, while smaller eigenvalues suggest greater dispersion. Therefore, eigenvalues can reveal the local dimensional features of the point cloud, as shown in Figure 5.

(1) If λ_1_ ≫ λ_2_ ≈ λ_3_, local features of the point cloud exhibit one-dimensional linearity.
(2) If λ_1_ ≈ λ_2_ ≫ λ_3_, local features of the point cloud exhibit two-dimensional plane dispersion.
(3) If λ_1_ ≈ λ_2_ ≈ λ_3_, local features of the point cloud exhibit three-dimensional sphere dispersion.

To better describe the relationship between eigenvalues and local dimensionality features, we construct a local dimensionality feature probability model (*L*_λ_, *P*_λ_, *S*_λ_) of point cloud based on Equation (5). The probability that a local point cloud belongs to different dimensional features is represented by the ratio of eigenvalue difference to the eigenvalue itself.

(5) $$\left\{\begin{array}{l} L_{\lambda }=\frac{\lambda_{1}-\lambda_{2}}{\lambda_{1}}\\ P_{\lambda }=\frac{\lambda_{2}-\lambda_{3}}{\lambda_{1}}\\ S_{\lambda }=\frac{\lambda_{3}}{\lambda_{1}} \end{array}\right.$$

where *L*_λ_, *P*_λ_ and *S*_λ_ represent the probabilities that the point cloud belongs to a one-dimensional linearity, two-dimensional plane, three-dimensional sphere, respectively. *L*_λ_ + *P*_λ_ + *S*_λ_ = 1. As shown in Equation (5), the probability of local dimensionality features of point clouds depends on neighborhood eigenvalues, while the neighborhood size significantly affects both eigenvectors and eigenvalues. To determine the optimal neighborhood radius, we introduce the theory of information entropy minimization [33]. A local entropy function for point clouds is constructed according to Equation (6), with the minimum entropy value serving as the constraint for determining the optimal neighborhood radius R.

(6) $$\left\{\begin{array}{l} H_{f_{i}}=-\left(L_{\lambda_{i}}\ln\left(L_{\lambda_{i}}\right)+P_{\lambda_{i}}\ln\left(P_{\lambda_{i}}\right)+S_{\lambda_{i}}\ln\left(S_{\lambda_{i}}\right)\right)\\ R_{\mathrm{excel}}^{i}=\mathrm{argmin}\left(H_{f_{i}}\right)R_{\mathrm{excel}}^{i}\in \left(R_{\min},R_{\max}\right) \end{array}\right.$$

where $H_{f_{i}}$ is the local neighborhood information entropy corresponding to the *i*th point, $R_{\mathrm{excel}}^{i}$ represents the optimal neighborhood radius corresponding to the minimum information entropy of the *i*th point, and (*R*_min_, *R*_max_) is the range of neighborhood radius.

**Figure 6:**
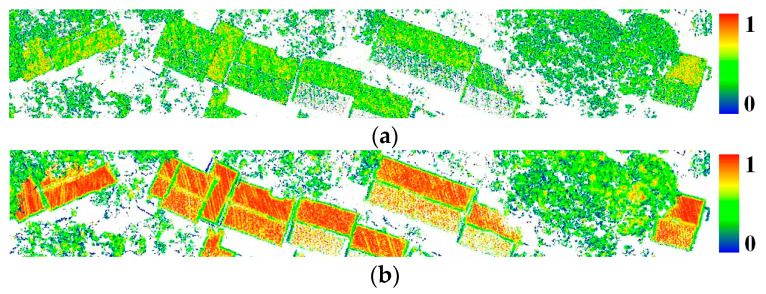
Two-dimension probability of point cloud: (**a**) unreasonable neighborhood radius; (**b**) optimal neighborhood radius.

Figure 6 illustrates the probability distribution of two-dimensional planar features of point clouds under optimal neighborhood radius and random neighborhood radius. Comparing Figure 6a,b, it is evident that an unreasonable neighborhood radius results in a lower probability of the building point falling into the two-dimensional plane. However, the optimal neighborhood radius accurately reflects that the probability of the building points falling into the two-dimensional plane is nearly 1. Additionally, the points close to the building edge and sparse in Figure 6b exhibit a low two-dimensional planar probability due to insufficient neighboring points. This issue will be addressed during subsequent morphological feature selection.

To show more clearly the feature probabilities and information entropy corresponding to different neighborhood radius, Table 2 displays the probability of local dimensionality under radius. By analyzing Equation (6) and Table 2, it can be seen that the smaller the information entropy value, the higher the probability of a certain dimension. Therefore, it makes sense to use the minimum information entropy to find out the optimal neighborhood radius of each point. Upon further analysis, it is obvious that an excessively large neighborhood radius leads to high information entropy. Since the purpose of this section is to find the minimum information entropy, continuously increasing the radius range would be meaningless. Therefore, the radius range is set between 0.1 and 1 m. Simultaneously, considering that computer performance struggles to handle the massive computational load resulting from overly small step length, it is set to 0.1 m.

#### 2.4.2. Analysis of Normal Vector Direction Consistency

The feature probability model extracted the majority of building points, but due to point cloud thickness and surface roughness, a portion of the point cloud remains unassigned to buildings. Generally speaking, building roofs are predominantly flat or sloped with a smooth surface. Consequently, laser points on the same roof surface often share similar characteristics, with greatly consistent normal vector direction among adjacent normal vectors. However, the vegetation canopy shows significant elevation variations with a complex, rough surface, resulting in highly variable normal vectors. Therefore, the consistency of normal vectors at adjacent points can be used to further filter building points.

Equation (7) is used to calculate the angle between adjacent normal vectors.

(7) $$\cos\theta =\frac{{\vec{N}}_{i}\cdot {\vec{N}}_{j}}{\left|{\vec{N}}_{i}\right|\cdot \left|{\vec{N}}_{j}\right|}$$

where ${\vec{N}}_{i}$,${\vec{N}}_{j}$ is the estimated normal vector of points *Q_i_* and *Q_j_*_,_ respectively.$\left|{\vec{N}}_{i}\right|$,$\left|{\vec{N}}_{j}\right|$ represents the length of ${\vec{N}}_{i}$,${\vec{N}}_{j}$,respectively. *θ* represents the angle between adjacent normal vectors.

**Figure 7:**
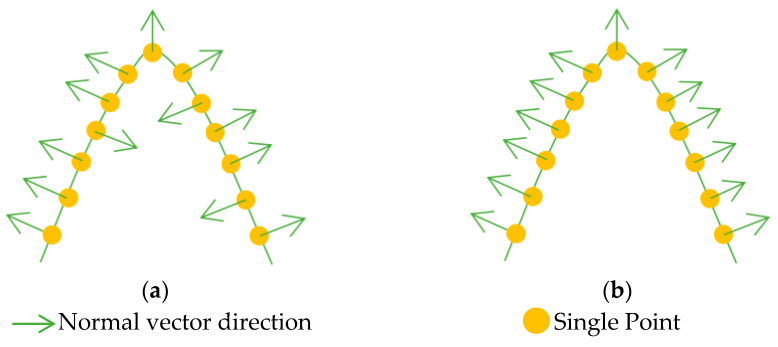
Schematic diagram of improving consistency in normal vector direction: (**a**) normal vector directions are various; (**b**) normal vector directions are consistent.

To ensure consistency in the direction of the normal vector, the direction is unified based on Equation (8), starting from the viewpoint direction.

(8) $$\left\{\begin{array}{c} {\vec{Q}}_{i}\cdot {\vec{Q}}_{j}>0\;\mathrm{No}\;\mathrm{change}\\ {\vec{Q}}_{i}\cdot {\vec{Q}}_{j}<0\;\mathrm{Reverse} \end{array}\right.$$

where ${\vec{Q}}_{i}$ represents the viewpoint vector of point *Q_i_*, which originates from the neighborhood center of *Q_i_* and terminates at *Q_i_*. Similarly, it follows that ${\vec{Q}}_{j}$. The improvement of normal vector direction consistency is shown in Figure 7.

**Figure 8:**
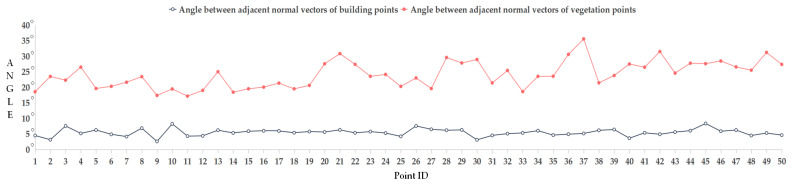
Direction consistency analysis of normal vector angles.

To clearly compare the differences in normal vector angles, we randomly selected 50 points each from the building point cloud and vegetation point cloud, calculated the normal vector angles between each point and its nearest point, and plotted the results as line-charts. As shown in Figure 8, it is evident that the normal vectors of the building points exhibit high consistency, with the angles between them generally falling within 10°. In contrast, the normal vectors of vegetation points are in different directions, showing an undiscernible pattern. It is evident that the table provided us with a clear angle threshold of 10° that was derived from 50 representative and persuasive random points. Therefore, subsequent experiments will not conduct sensitivity tests for this angle threshold.

#### 2.4.3. Random Sample Consensus Plane Fitting

**Figure 9:**
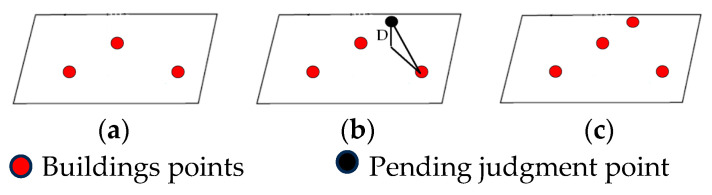
The iteration procedure of RANSAC: (**a**) initial plane parameter estimation; (**b**) iteration procedure; and (**c**) plane parameter refined.

To further address misclassification of building points that are close to the roof’s edge, the Random Sample Consensus (RANSAC) [28] is adopted to estimate parameters of plane from locally clustered building points. As shown in Figure 9, we randomly select three points from the building point to obtain the initial plane parameter. Then, by evaluating the perpendicular distance D between points and planes, new building points are continuously incorporated to refine the plane parameters until iteration stops.

During the procedure above, the iteration count *k* is a critical parameter to achieve the optimal plane parameters, which is calculated by Equation (9).

(9) $$k=\frac{\ln\left(1-p\right)}{\ln\left(1-w^{n}\right)}$$

where *w* represents the probability of selecting any single in-cluster point from a given clustered point cloud. *w*^n^ denotes the probability of selecting *n* points, all of which are in-cluster points. 1 − *w*^n^ indicates the probability that at least one of the selected n points is an out-of-cluster point. *p* signifies the probability that the selected subset contains at least one instance where all points are in-cluster.

## 3. Experiments and Discussion

Three groups of point clouds with different building distribution and various terrain types were selected to verify the practicality and accuracy of the method proposed in this paper.

### 3.1. Experimental Datasets

**Figure 10:**
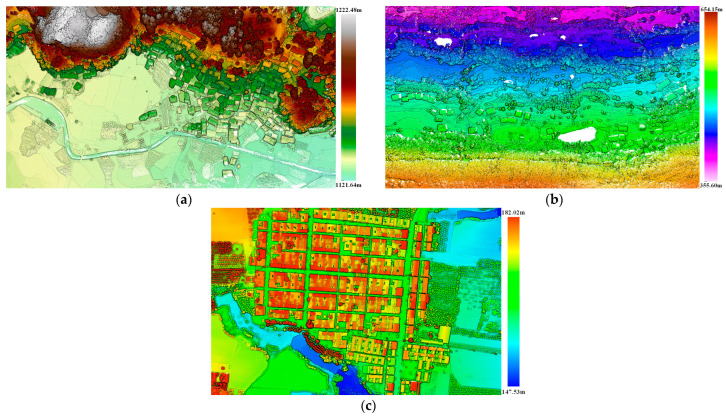
Details of experimental datasets: (**a**) Dataset 1; (**b**) Dataset 2; and (**c**) Dataset 3.

Dataset 1 and Dataset 2 were collected using the XT16 laser scanner from HESAI Technology Company in China; the flight platform is a multi-rotor UAV with an altitude of approximately 150 m. Dataset 3 was collected by the VUX-1LR-22 laser scanner from Riegl, and the flight platform is a fixed-wing UAV with an altitude of approximately 400 m. The details of the datasets are listed in Table 3. As shown in Figure 10, Dataset 1 features plains and mountainous terrain with a maximum elevation difference of 100.84 m. Buildings are dense and concentrated in a terraced pattern along the mid-slopes. Dataset 2 also mainly contains plains and mountains with a maximum elevation difference of 298.55 m, but buildings are predominantly clustered on flat land. Dataset 3 represents a flatland area with an elevation difference of 34.49 m. Buildings are predominantly single-story structures and exhibit a highly concentrated distribution. These datasets exhibit different building densities, distribution, and roof styles, comprehensively representing typical rural building features.

### 3.2. Evaluation Methods

This section defines the methods used for calculating the accuracy of building classification. The manually classified building points are used as the truth, which is based on the criterion that the ground appears visually smooth to the eye with no obvious irregular protrusions or discontinuities. This aligns with actual data processing experience and the evaluation criteria outlined in Reference [34]. The precision–recall method [34] is used to quantitatively evaluate the effectiveness and accuracy of point cloud classification. These are defined as follows:

(10) $$\left\{\begin{array}{l} \mathrm{Precision}=\frac{TP}{TP+FP}\\ \mathrm{Recall}=\frac{TP}{TP+FN}\\ F1=2\cdot \frac{\mathrm{Precision}\cdot \mathrm{Recall}}{\mathrm{Precision}+\mathrm{Recall}} \end{array}\right.$$

where *TP*, *FP*, and *FN* represent the quantities of true-positive, false-positive, and false-negative results relative to the building truth, respectively. Generally, *TP* and *FP* are mutually contradictory and constrain each other. To provide a more comprehensive evaluation of classification accuracy, F1 is introduced as a synthetic indicator to balance the influence of precision and recall.

### 3.3. Experiment Parameters

The method proposed involves three sections: ground point filter, ROI selection of building, and refined building classification. To more clearly demonstrate the applicability of this method, Table 4, Table 5 and Table 6 list point cloud classification parameters which are suitable for most scenarios.

The ground filter is related to primary grid size, iteration distance, and angle. First, the minimum length of a building is typically within 15 m. To ensure that the secondary grid size adequately encompasses the minimum length of buildings and to prevent buildings from being incorrectly classified as ground, the primary grid size should be no less than twice that of the secondary grid, meaning the initial grid size should be 30 m. Second, for flat terrain, the iteration angle should not be too large, with 3–5° being optimal, and the iteration distance set at 0.5 m. For hilly regions, an iteration angle of 7–9° and an iteration distance of 1.2–1.5 m are recommended. For mountainous areas, an iteration angle of 10–12° and an iteration distance of 1.4–1.8 m should be adopted.

**Figure 11:**
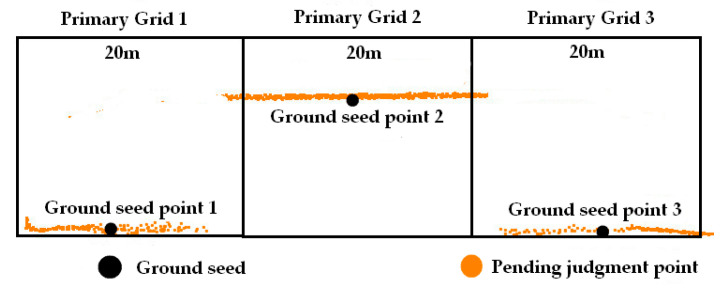
Sensitivity analysis of primary grid size.

For analyzing the sensitivity of primary grid size, we adopted 20 m as the primary grid size to filter the ground. As can be seen in Figure 11, building roofs are erroneously classified as ground points in some regions. This occurs because the primary grid is too small, preventing the secondary grid from fully encompassing the building, so roof points are mistakenly selected as ground seed points. In comparison, ground point filtering with a primary grid size of 30 m avoids this issue. This is mainly due to the 30 m grid being able to encompass most of the buildings.

Regarding the iteration distance and angle, we employed a commonly suggested parameter according to research [26,29] and extensive engineering experience, which had a reasonable range of parameters suitable for various terrains. Therefore, we will not further discuss the sensitivity of these parameters.

The building ROI selection is related to maximum height difference, standard deviation of height, and projected area. The typical height of one floor is approximately 3 m, so the maximum elevation difference threshold should be 9 m. To prevent low vegetation from affecting building ROI selection, we stipulate that the maximum elevation difference should exceed 2 m. The segmentation area includes buildings, the ground, and vegetation, and typically falls within the median range of these three components, so the standard deviation is set at 3–6 m. Typically, building areas generally exceed 20 m^2^, while vegetation canopy areas vary, and young plant canopy areas are generally less than 2 m^2^.

Building refined extraction is related to dimensional feature probability, directional consistency of vector normal, and plane fitting. Dimensional feature probability relies on the neighborhood radius size, which is determined by the minimum information entropy automatically. The consistency of normal vectors is determined by the angle between them, with a threshold of less than 10°. Plane fitting is determined based on iteration count and perpendicular distance. Commonly, iteration count is 1000 and perpendicular distance depends on the thickness of the point cloud used in the experimental dataset, which is approximately 5 cm. 

### 3.4. Experiment Results

In this section, the three datasets above with different terrain and building distribution are used to conduct the experiment. To clearly demonstrate the effectiveness of ROI filtering, we listed the total amount of experimental data point clouds, the total number of point clouds within the ROI, and the removal rate. As shown in Table 7, ROI filtering can exclude approximately 70% of non-building points in advance, thereby reducing unnecessary computations to a certain extent.

**Figure 12:**
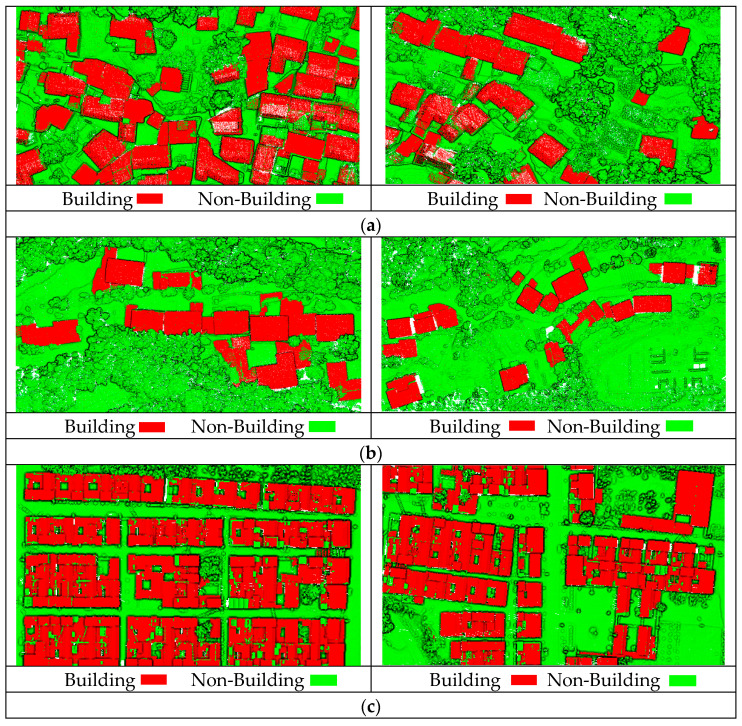
The effect of building classification method proposed in this paper: (**a**) the effect of building classification for Dataset 1; (**b**) the effect of building classification for Dataset 2; and (**c**) the effect of building classification for Dataset 3.

Figure 12 shows the effect of building classification; the overall classification effect of the three groups’ experimental datasets is extremely positive. Regardless of whether we applied the building classification procedure to low-rise residential buildings or complex building roofs, no large-scale omissions of building structures happened. Although some buildings are adjacent to vegetation or backed by mountains, making them susceptible to interference from external factors, the buildings exhibit continuous and compact geometric forms with complete shapes. Building point clouds are effectively identified, while non-building points such as trees are efficiently filtered out. However, a small portion of other objects with similar heights to buildings, such as temporarily parked box trucks, were identified as buildings. This is another issue that needs to be addressed in the future.

Table 8 quantitatively analyzes the accuracy of the building classification method proposed in this paper. For Dataset 1, the precision, recall, and F1 values were 93.37%, 97.05%, and 95.17%, respectively. For Dataset 2, the precision, recall, and F1 values were 94.08%, 97.22%, and 95.62%, respectively. For Dataset 3, the precision, recall, and F1 values were 94.61%, 97.33%, and 95.95%, respectively. This demonstrates that the building classification method proposed achieves both high precision and high recall when facing complex scenarios, with both values above 93.37%. It not only identifies structures accurately but also comprehensively, maintaining consistently high and stable building classification accuracy. The algorithm exhibits great applicability and high practical value for engineering applications.

### 3.5. Comparisons

To compare the accuracy and applicability of different building classification methods, this paper introduces other methods [9,34] for comparison with the method proposed in the thesis. As shown in Table 9, the accuracy and recall of the building classification method proposed in this paper outperforms the other two methods. This indicates that the proposed method achieves higher precision and recall for positive samples, demonstrating greater applicability for building classification across diverse complex scenarios.

The above results are closely related to the philosophy of methods. Method A [6] relies entirely on the DSM derived from point cloud. Although this method establishes a series of feature indicators and combines maximum likelihood classification to extract buildings, it sacrifices some of the point cloud’s inherent accuracy, resulting in the poorest overall precision. Method B [26] integrates point cloud feature differences and density characteristics within voxels to construct adaptive weights, optimizes the building seed point selection strategy, and finally completes building classification through clustering. When confronted with interlocked vegetation and buildings, this method is prone to selecting incorrect seed points to some extent, thereby affecting the final classification results. In contrast, the method proposed in this paper fully uses multi-dimensional feature differences to progressively achieve building classification. Nevertheless, it is certain that the philosophies of the other two methods remain worthy of reference.

## 4. Conclusions

According to the features of terrain and building in rural areas, we developed a building classification method based on airborne point clouds, which possesses a strong sense of spatial hierarchy, comprehensively considering the spatial distribution characteristics of buildings to exclude partial non-building points by ROI filtering. At the same time, it consistently focuses on the fundamental differences in geometric and morphological features between buildings and other objects, extracting building data from coarse to fine. Three datasets with various building heights and styles and distinct building densities and spatial distribution are used to verify and examine the accuracy and practicality of the methodology presented in this paper. Quantitative analysis results indicated that the precision, recall, and F1 values for both datasets exceeded 93.37%, 97.05%, and 95.17%, respectively. The average precision, recall, and F1 scores reached 94.02%, 97.20%, and 95.58%, respectively. This demonstrated that the building classification method shows high accuracy and good stability, which facilitates rapid building classification in large rural regions and serves subsequent practical applications, such as 3D reconstruction, urban management and infrastructure planning.

This method performed moderately when classifying regular-shaped objects similar in height to buildings, such as trucks. In addition, we did not consider how to ensure building classification accuracy when point cloud density is insufficient. In further research, we will fully consider integrating multi-sources data such as airborne LiDAR point clouds, aerial imagery, and multispectral imagery with deep learning to extract higher-level abstract features to enhance the universality and applicability of this method.

## Figures and Tables

**Table 1:** Types of terrain.

ID	Types	Range of Slope
1	Flat	0–10°
2	Hilly	10–20°
3	Mountain	>20°

**Table 2:** Dimensional feature probability and information entropy of points under different radii.

Radius	Information Entropy	Probability of Being One-Dimension	Probability of Being Two-Dimension	Probability of Being Three-Dimension
0.1 m	0.579	0.825	0.064	0.111
0.2 m	0.931	0.561	0.108	0.331
0.3 m	1.050	0.441	0.198	0.361
0.4 m	1.033	0.187	0.335	0.478
0.5 m	1.037	0.301	0.491	0.208
0.6 m	0.611	0.216	0.765	0.019
0.7 m	0.142	0.017	0.973	0.010
0.8 m	0.536	0.082	0.846	0.072
0.9 m	0.792	0.219	0.697	0.084
1.0 m	0.998	0.137	0.433	0.430

**Table 3:** Details of datasets.

ID	Lowest	Highest	Height Difference	Type of Terrain	Building Density	Building Distribution
1	1121.6 m	1222.48 m	100.84 m	Plain/Mountain	Dense	Mid-slopes
2	355.60 m	654.15 m	298.55 m	Plain/Mountain	Sparse	Plain
3	147.53 m	182.02 m	34.49 m	Plain	Dense	Plain

**Table 4:** Ground filter parameters.

Type of Terrain	Primary Grid Size	Iteration Distance	Iteration Angle
Flat	30 m	0.5 m	3–5°
Hilly		1.2–1.5 m	7–9°
Mountains		1.4–1.8 m	10–12°

**Table 5:** Parameters for building ROI selection.

Maximum Height Difference	Standard Deviation of Height	Projected Area
2–9 m	3–6 m	≥20 m^2^

**Table 6:** Parameters for building refined extraction.

Dimensional Feature Probability	Angle Threshold	Iteration Count	Perpendicular Distance
Determined Automatically	≤10°	1000	≤5 cm

**Table 7:** Comparison of point cloud quantity before and after ROI filtering.

Dataset	Total Amount of Point Clouds	Total Amount of Point Clouds Within ROI	Removal Rate
Dataset 1	116,676,854	34,256,325	70.64%
Dataset 2	74,042,797	20,458,025	72.37%
Dataset 3	19,774,769	5,287,774	73.26%

Note: The removal rate is the ratio of the difference between total amount of point clouds and total amount of point clouds within ROI to total amount of point clouds.

**Table 8:** Accuracy of building classification method.

	Classification	Building	Non-Building	Precision	Recall	F1
Dataset 1	Building	5,818,009	413,124	93.37%	97.05%	95.17%
	Non-Building	176,848	110,268,873			
Dataset 2	Building	4,094,932	257,674	94.08%	97.22%	95.62%
	Non-Building	117,094	69,573,097			
Dataset 3	Building	3,949,391	224,999	94.61%	97.33%	95.95%
	Non-Building	108,341	15,492,038			

**Table 9:** Comparison of three methods.

Dataset	Accuracy Index	Method Proposed	Method A [9]	Method B [34]
Dataset 1	Precision	93.37%	92.11%	93.21%
	Recall	97.05%	94.25%	95.17%
	F1	95.17%	93.27%	94.18%
Dataset 2	Precision	94.08%	91.60%	92.26%
	Recall	97.22%	94.27%	93.91%
	F1	95.62%	92.92%	93.08%
Dataset 3	Precision	94.61%	92.86%	94.05%
	Recall	97.33%	95.37%	96.64%
	F1	95.95%	94.10%	95.33%

## Data Availability

The data used to support the findings of this study are available from the corresponding author upon request.

## Abbreviations

The following abbreviations are used in this manuscript:

LiDAR	Light Detection and Ranging
PTD	Progressive Triangulated Irregular Network Densification Filtering
CSF	Cloth simulation filtering
TIN	Triangulated Irregular Network
DTM	Digital Terrain Model
NDSM	Normal Digital Surface Model
DSM	Digital Surface Model
ML	Machine Learning
SC-BOVW	Sparse Coding-enhanced Bag of Visual Word
SVM	Support Vector Machine
DL	Deep Learning
PTDTA	Progressive TIN Densification of Terrain Awareness
ROI	Region of Interest
PCA	Principle Component Analysis
RANSAC	Random Sample Consensus
